# Gossypiboma during orthognathic surgery: A case report

**DOI:** 10.1016/j.ijscr.2020.09.117

**Published:** 2020-09-23

**Authors:** Saleh Zaid Alshehri, Mohammed Ghazi Alkindi, Osama A. Ibraheim, Razan A. Ababtain, Randa Alfotawi

**Affiliations:** a Residant at Department of Oral and Maxillofacial Surgery, College of Dentistry, King Saud University, Riyadh 11433, Saudi Arabia; b Department of Oral and Maxillofacial Surgery, Faculty of Dentistry, King Saud University, P.O. Box 60169, Riyadh 11545, Saudi Arabia; c Department of Anesthesiology, College of Medicine, King Khalid University Hospital, King Saud University, Riyadh 11433, Saudi Arabia; d Residant at Maxillofacial Surgery, National Guard for Health Affairs, Riyadh, Saudi Arabia

**Keywords:** Aspiration, Hazard, Orthognathic surgery, Temperature probe, Complications, Gossypiboma, Unretrieved device fragment (UDF)

## Abstract

**Introduction:**

Dislodgment of nasopharyngeal temperature probes and/or unretrieved device fragments (UDFs) or gossypibome at a patient’s hypopharynx is rare complication after orthognathic surgery that may occur as a result of surgical manipulation or may be a consequence of factors related to the insertion and handling of the probe after extubation. However, the exact mechanism of this complication is unknown. To the best of our knowledge, this is the 1st reported case of a missing temperature probe after orthognathic surgery.

**Case presentation:**

We report the case of a patient who suffered from dislodgment of a 12-cm temperature probe after orthognathic surgery. The surgery was uneventful. At the end of the surgery, the probe was believed to have been completely removed from the nasal cavity. The nasopharyngeal cavity was visually inspected while the patient was still under anaesthesia and the trachea was still intubated. Extubation was successful, and the patient was moved to the recovery area. The patient was discharged from the hospital one day after resuming an oral fluid diet. At the follow-up visit on the 4th postoperative day, the patient presented with mild symptoms of a sore throat and cough. At the follow-up visit in the 3rd postoperative week, the patient reported one episode of vomiting and severe coughing, and the patient ultimately retrieved the 12-cm temperature probe from her mouth.

**Discussion:**

After conducting a systematic literature review, we discuss surgical cases involving UDFs or gossypiboma. We also describe changes in our clinical practice after this event, and we envision that these modifications will have a positive influence on patient care. We believe that alternative routes for inserting temperature probes with covers would be suitable for orthognathic surgery.

**Conclusion:**

Vigilance should be maintained during patient extubation by both teams (surgeons and anaesthetists) to assure that part of the probe always remains visible outside the oral/nasal cavity as well as complete removal of the device to avoid this life-threating complication.

## Highlights

• An unusual case of Unretrieved Device Fragment (UDF) at patient’s hypopharynx for 21 days.
• First reported complication after orthognathic surgery.
• This incidence has changed the routine anaesthetic procedure protocol.
• Update our time out check list of to include “complete removal of temperature probe.

## 1 Introduction

Maxillofacial surgery presents many challenges for anaesthesiologists. A primary challenge is the shared airway and the presence of anaesthetic equipment virtually within the surgical field. A wide variety of postoperative complications have been reported to accompany orthognathic surgery and have led to many problems in many cases [1]. Most of these complications can be managed through proper treatment and with a sufficient understanding of their causes, but some complications are unusual and difficult to predict [2]. An unretrieved device fragment (UDF) or gossypiboma of the nasopharyngeal temperature probe after surgery is a rare complication [3]. However, to the best of our knowledge, this is the 1st reported case of a missing temperature probe after orthognathic surgery.

The term gossypiboma or UDF is a technical term for surgical complications resulting from foreign materials such as surgical sponges accidentally left inside a patient’s body. The incidence of gossypiboma has been reported more frequently in abdominal surgery at a rate of 1:3000 operations 4, whereas the incidence of UDF among all surgeries has been reported to be 0.01% to 0.001%, with 80% of cases being gossypibmas [4]. Two main consequences of having a UDF have been documented to be secondary bacterial infection and foreign body granuloma. Symptoms of odour may not present for a long time, sometimes appearing even months to years later [4,5].

The standards for monitoring patients during anaesthesia and recovery recommend continuous core temperature monitoring because intraoperative hypothermia can result in serious adverse effects such as myocardial ischaemia, coagulopathy, and surgical wound infection. Nasopharyngeal temperature probes are commonly used for temperature monitoring during general anaesthesia and can serve as an alternative to oesophageal probes when appropriate. The advantages of nasal probes are their cost-effectiveness and their ability to measure core temperature. However, some authors claim that the use of an oropharyngeal probe is more expensive than a nasopharyngeal probe because the latter requires adequate mucosal attachment to accurately measure the core temperature [8].

Orthognathic surgery is recognized as a safe and predictable procedure due to an increase in the knowledge of facial anatomy, and progress in orthodontic treatment over the last 30 years has resulted in low morbidity associated with this procedure [1,7]. Initially, maxillofacial orthopaedic surgery was intended for the treatment of malocclusion in younger populations; however, with the development of this surgery, anaesthesia techniques also changed and became more sophisticated, thus allowing the surgery to be performed for patients across different age groups with various craniofacial anomalies.

We report an unusual case of a nasopharyngeal temperature probe UDF in a patient’s hypopharynx for 21 days following an uneventful elective orthognathic surgery. Although no serious morbidity was observed in our patient, this case resulted in several changes to the guidelines for this routine anaesthetic procedure in our clinical practice.

## 2 Case report

A healthy 23-year-old female presented with increased gingival display (>7 mm) during maximum smiling. She was diagnosed with a class II skeletal dentofacial deformity due to an increase in the lower facial height and a high mandibular angle. She had no significant medical problems in the past. No drug history or relevant genetic or psychological abnormalities were reported. Upon physical examination, the patient had a Mallampati-I airway, and her mental-hyoid distance and mouth opening were normal. She weighed 71 kg and was 1.78 m tall (BMI, 22.4 kg/m^2^). Intraorally, she presented with generalized mild periodontal Class II dental occlusion. Preoperative evaluation revealed that her blood pressure was 110/80, and her heart and lung examinations (electrocardiogram and chest radiography) were normal.

A single jaw osteotomy was planned for the case, resulting in a maxillary differential impaction of 6 mm anteriorly with partial inferior turbinectomy, and autorotation was applied to the mandible to correct her mandibular prominence.

Preoperatively, in the waiting area, the patient received 2 mg of midazolam. After routine monitoring, rapid sequence induction of general anaesthesia was performed with intravenous fentanyl, lidocaine, and propofol. Following tracheal intubation, a 9 French thermistor temperature probe (Model VER400-9, VHA Inc., Irving, TX, USA) was inserted approximately 12 cm into the nasopharynx.

The surgery started by marking the reference point using a K-wire near the nasal bone. Then, consecutive measurements were taken from the orthodontic brace to the reference point. Then, two incisions were made 6 mm superior and inferior into the maxillary bone at the left I level. The incisions were achieved using a surgical saw and a handheld motor (Stryker, Germany). The incisions started at the lateral nasal wall, passed the pyriform area, extended back to the pterygomaxillary buttress and reached the junction.

This step was followed by a maxillary down-fracture with a pterygomaxillary disjunction using a curved Obwegeser osteotome; no difficulties or injuries occurred. An inferior turbinectomy was performed bilaterally. After achieving the desired occlusion guided by a surgical stent, the bone was fixed using two plates and 6 screws on each side at the buttress (pyriform and pterygomaxillary). A cinch suture was placed at the alar base to maintain the inter alar distance, followed by soft tissue suture for the surgical sites using 3/0 Vicryl suture material.

After maintenance inhalational anaesthesia was stopped, the patient was given 100% oxygen. The throat pack was removed by the surgeon with proper Yankauer suctioning of the oropharynx. The surgery was performed by a senior consultant and senior and junior residents in the department. The patient was extubated, and the shortened nasopharyngeal temperature probe was not observed upon removal from the other nostril. The patient was transferred to another ward without any complications.

On the second postoperative day, the patient started to complain of mild throat discomfort on the lateral side and occasional cough. She was encouraged to follow an oral fluid diet. The patient tolerated the surgical procedure well and was discharged on the 2nd day after surgery without any other complaint except for mild cough and throat discomfort. After one week, the patient returned with increasingly severe symptoms of cough and throat pain, which were very irritating to the patient. The patient was referred for endoscopic evaluation to explore her pharynx. The endoscopic report was negative and showed a normal supraglottic area and an unremarkable laryngeal examination.

**Fig. 1:**
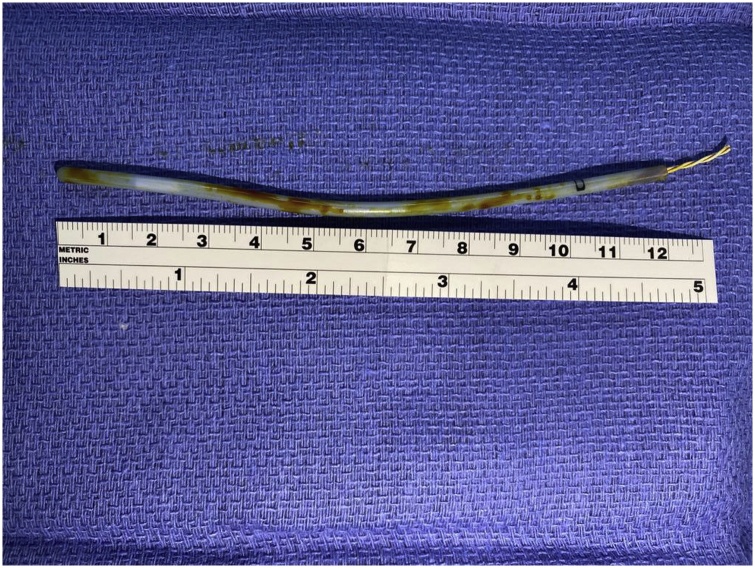
Severed temperature probe (12 cm long).

The patient was monitored closely with a frequent follow-up. At two weeks postoperatively, the patient was interviewed in the outpatient clinic, and she still complained of mild discomfort in her neck while speaking and inhaling, and she described feeling a foreign body. Three weeks after surgery, the patient started regular food habits, and the discomfort persisted. She reported one attack of a severe cough and concomitant vomiting where she noticed that a long plastic-covered object came out of her mouth (Fig. 1). Close inspection of the item confirmed our diagnosis; it had a bevelled cut end and was 12 cm long, indicating that it was part of the nasopharyngeal temperature probe. The inspection confirmed that it had been cut during the inferior turbinectomy procedure and that it was probably dislodged in the lateral pharyngeal wall for a long time without any remarkable gag reflex.

The patient immediately felt relief from the irritating symptoms in her throat. The patient was sent for PA chest X-ray radiography, which showed no remarkable findings. The patient’s follow-up course was uneventful, and she was satisfied with the result of the surgery. She was discharged after an 18-month follow-up period.

## 3 Patient consent

Written informed consent was obtained from the patient for publication of this case report and accompanying images. The work has been reported in line with the SCARE 2018 criteria [8].

## 4 Discussion

We report an unusual case of a temperature probe severed during orthognathic maxillary osteotomy and turbinectomy procedures. Our case is unique because of the late presentation of the patient, which led us investigate the possible mechanism responsible for our observations. The probe was likely dislodged within the lateral pharyngeal wall away from the gag reflex centre, thus causing mild symptoms and leading the surgeon to believe that the symptom was normal throat discomfort following general anaesthesia. The persistent cough and discomfort after 1 week were alarming, and the patient was referred for endoscopic exploration, which appeared to be negative. X-ray radiography would have been an ideal investigation tool in this clinical scenario.

One may wonder why the patient’s gag reflex was not activated and why she did not choke. The possible pathophysiology in this case is that the probe reached the patient’s throat pack and dislodged beyond the sensory nerve ending area (an area supplied by the glossopharyngeal nerve, which includes the back of the tongue, the area around the tonsils, the uvula, and the back of the throat) after the throat pack was removed but before the patient’s reflexes returned to normal after the anaesthetic effects subsided. Then, the temperature probe dislodged in the hypopharynx in the lateral pharyngeal wall, which is characterized by thick, deep, folded mucosa that complicate exploration even with endoscopy examination as in our case. A similar incidence of UDFs without symptoms was reported by Mishra et al. who reported an impacted metallic foreign body in the left lateral pharyngeal wall just above epiglottis [9]. Another asymptomatic 5-mm metallic object was observed in the retropharyngeal area and treated conservatively [10]. Ramdas et al. reported a case of a migrating intramural foreign body in the oesophagus [11]. The patient had a history of a foreign body in the aerodigestive tract, and radiologically, an open safety pin was found in the upper oesophagus. Endoscopy was performed and was found to be normal.

The other possible explanation is that this patient lacks a gag reflex with an intact reflexive pharyngeal swallow. A high incidence rate (37%) related to an absent gag reflex has been reported among patients, although they have an intact pharyngeal sensation [12]. One can infer that patients may have normal muscle function for swallowing, which is independent from the muscles controlling the gag reflex. Interestingly, the swallowing reflex also acts as protective mechanism for the upper respiratory system, forcing the glottis to close and clearing the pharynx from residual food substances [12]. Our patient did not undergo further investigation regarding her pharyngeal sensation.

According to a literature search, the reported incidence of temperature probe hazards is low; one article reported the case of accidental misplacement of the disposable plastic cover of a temperature probe in the nasopharyngeal cavity upon endotracheal extubation [13]. The cover was uneventfully extracted intact using a sinus scope, nasal speculum, and bayonet forceps [13]. Another article reported that the first nasopharyngeal temperature probe was dislodged during elective revision laparoscopic Nissen fundoplication ligature, and the probe was retrieved immediately before the patient left the operating room [3]. Last, a case of severe bleeding from the nostril of a patient on anticoagulants was reported due to improper handling of a temperature probe, which caused injury to the nasal mucosa [14].

As a result of this event, several changes were made to our anaesthetic and surgical practices to prevent this complication. First, we have augmented our time-out check list with the phrase “complete retrieval of the temperature probe” for safer practice as part of the operating room protocol. We intend to educate employees by reporting this case, warn employees about the potential for this complication during maxillofacial surgery, and teach them how to avoid such complications. Second, for patients with indwelling urinary catheters, we suggest that core temperature should be monitored using a bladder probe (e.g., Model Bardex® Lubricath Temperature- Sensing Foley Catheter, C.R. Bard, Inc., Covington, GA, USA). Alternatively, when nasopharyngeal temperature monitoring is necessary, we suggest using a temperature probe that is sufficiently short to avoid hypopharyngeal or even oesophageal dislodgment during bougie dilator insertion [3]. However, studies have shown that more than half of blindly placed nasopharyngeal temperature probes in clinical practice are suboptimal [15]. Alternatively, using oropharyngeal probes has been discussed in the literature and can be a safe option, especially in orthognathic surgery. Moreover, using oropharyngeal probes that show substantial temperature differences in the nasal cavity or oropharynx compared with the upper nasopharynx has been reported to be advisable [6]. Therefore, superficial insertion of a probe into the nasal cavity is less accurate than deep insertion of a probe into the oropharynx if the probe is not placed optimally at the upper nasopharynx [6].

Third, the surgical and anaesthesia teams should communicate to ensure that all foreign bodies have been withdrawn from the mouth and nose except for the endotracheal tube and the oesophageal bougie. If any doubt remains, chest X-ray may be advisable before patient extubation.

In summary, we report the first case of nasopharyngeal temperature probe entrapment during elective orthognathic surgery that precipitated a continuous quality improvement project at our institution. We envision that the above changes to our clinical practice will have a positive influence on patient care and should be considered when caring for this surgical patient population.

## Declaration of Competing Interest

There is no conflict of interest over this paper.

## Funding

No fund for this paper.

## Ethical approval

The case report was normal clinical operating case not part of any research studies.

## Consent

“Written informed consent was obtained from the patient for publication of this case report and accompanying images. A copy of the written consent is available for review by the Editor-in-Chief of this journal on request.”

## Author contribution

Saleh Shehri, Residant collecting the data.

Razan Babtain,Residant Surgery assisting.

Mohamad Kindi, Consultant on charge.

Osama Ibrahim, anaesthetic consultant.

Randa Alfotawi, Wrote the manuscript and corresponding author.

## Registration of research studies

1. an unusual case of a nasopharyngeal temperature probe dislodged in the patient’s hypopharynx for 21 days.
2. First reported complication after orthognathic surgery.
3. Changes the routine anaesthetic procedure protocol.

## Guarantor

RANDA ALFOTAWI

Assistant professor Oral and Maxillofacial Surgery

Dental department, King Saud University

e.amil:ralfotawei@ksu.edu.sa

## Provenance and peer review

Not commissioned, externally peer-reviewed.
